# Role of Sensorimotor Cortex in Gestural-Verbal Integration

**DOI:** 10.3389/fnhum.2018.00482

**Published:** 2018-12-06

**Authors:** Dayana Hayek, Agnes Flöel, Daria Antonenko

**Affiliations:** ^1^Charité—Universitätsmedizin Berlin, Corporate Member of Freie Universität Berlin, Humboldt-Universität zu Berlin, and Berlin Institute of Health, Department of Neurology, NeuroCure Clinical Research Center, Berlin, Germany; ^2^Department of Neurology, Universitätsmedizin Greifswald, Greifswald, Germany

**Keywords:** brain stimulation, embodiment, gestural-verbal association, cognition, language processing

## Abstract

Action comprehension that is related to language or gestural integration has been shown to engage the motor system in the brain, thus providing preliminary evidence for the gestural-verbal embodiment concept. Based on the involvement of the sensorimotor cortex (M1) in language processing, we aimed to further explore its role in the cognitive embodiment necessary for gestural-verbal integration. As such, we applied anodal (excitatory) and sham transcranial direct current stimulation (tDCS) over the left M1 (with reference electrode over the contralateral supraorbital region) during a gestural-verbal integration task where subjects had to make a decision about the semantic congruency of the gesture (prime) and the word (target). We used a cross-over within-subject design in young subjects. Attentional load and simple reaction time (RT) tasks served as control conditions, applied during stimulation (order of three tasks was counterbalanced). Our results showed that anodal (atDCS) compared to sham tDCS (stDCS) reduced RTs in the gestural-verbal integration task, specifically for incongruent pairs of gestures and verbal expressions, with no effect on control task performance. Our findings provide evidence for the involvement of the sensorimotor system in gestural-verbal integration performance. Further, our results suggest that functional modulation induced by sensorimotor tDCS may be specific to gestural-verbal integration. Future studies should now evaluate the modulatory effect of tDCS on semantic congruency by using tDCS over additional brain regions and include assessments of neural connectivity.

## Introduction

The engagement of the sensorimotor system in word comprehension has been an intriguing question in brain research (Hauk et al., 2004; Tettamanti et al., 2005; Pulvermuller and Fadiga, 2010; Vukovic et al., 2017). Gestures were found to enhance language comprehension of listeners (Hostetter, 2011), possibly via embodiment. The embodiment concept, also referred to as grounded cognition, is based on involuntary mimicry (Barsalou, 2008). Parzuchowski et al. (2014) used embodied cognition to show that hand gestures enhance language comprehension. According to Hostetter and Alibali (2008), the embodiment approach suggests that language understanding is based on perceptual experience, that is, words start to have meaning when linked to real world perception.

Gestural-verbal integration has been studied at both the behavioral and the neural level. It has been suggested that language comprehension is facilitated by gestures due to the presence of common neural substrates for processing language and gesture (Holle et al., 2008; Dick et al., 2009; Hubbard et al., 2009; Straube et al., 2012). These neural substrates include both inferior frontal gyrus (IFG; Straube et al., 2011; Dick et al., 2014; He et al., 2018b) and motor cortex (for review Ozyurek, 2014). Studies have reported activation in the primary motor cortex during word comprehension when words involved sensorimotor features (Willems and Hagoort, 2007; Pulvermuller and Fadiga, 2010), using various methodological approaches. These approaches include functional magnetic resonance imaging (fMRI; Kemmerer et al., 2008; Kana et al., 2012, 2015), electroencephalography (Mollo et al., 2016; Schaller et al., 2017) and magnetoencephalography (Klepp et al., 2015; Mollo et al., 2016). The findings have raised the intriguing question why language comprehension is processed in a sensorimotor area (De Marco et al., 2015). One explanation might be that mirror neurons are responsible for this interaction (Caramazza et al., 2014). Aridan and Mukamel (2016) found that observing someone else performing a task enhances one’s own performance. Simultaneously acquired fMRI data showed a significant positive correlation between blood oxygen level dependent (BOLD) fMRI response within left sensorimotor cortex (M1) during action observation and the execution rate of the subjects. Thus, activation of the same fronto-parietal sensorimotor areas in both observing and executing an action enables an individual to understand the observed action more easily (Rizzolatti et al., 2001).

In addition to neuroimaging, non-invasive brain stimulation is a promising approach to investigate the involvement of the stimulated cortex in the respective function (Jacobson et al., 2012; Parkin et al., 2015; Tremblay et al., 2016; Polania et al., 2018). Moreover, stimulation may modulate performance in the task under study. In particular, transcranial direct current stimulation (tDCS) was found to modulate cognitive functions and underlying neuronal activity and connectivity (Meinzer et al., 2012; Parkin et al., 2015; Lavidor, 2016; Strobach and Antonenko, 2017; Yavari et al., 2017). More recent studies have used tDCS to reveal a role for M1 in language comprehension, especially for action-related words (Meinzer et al., 2016; Branscheidt et al., 2018). These results provided evidence for an interaction of motor cortex activity with language processing. A more recent study found that for healthy individuals, anodal tDCS (atDCS) over the motor cortex improved semantic word retrieval performance (Martin et al., 2017). In terms of gesture comprehension, studies have used tDCS over the IFG to investigate the role of this region in processing gestural-verbal stimuli (Cohen-Maximov et al., 2015; Schulke and Straube, 2017, 2018). Gesture prime clips were implemented for word targets. Participants were instructed to make a semantic decision of the prime-target congruency. Subjects responded faster under atDCS of the right IFG compared to sham tDCS (stDCS). The study suggested that inferior frontal atDCS may enhance gestural-verbal integration, which in turn enhances gesture comprehension. However, no previous study, to our knowledge, explored the role of left M1 for gestural-verbal integration, using tDCS.

In our study, we therefore assessed the impact of atDCS over left M1 on gestural-verbal integration (adapted from Cohen-Maximov et al., 2015). In order to exclude that the effect on gestural-verbal task would be based on improved attentional and motor processes, we included two control tasks during stimulation [attentional load task and simple reaction time (RT) task]. Given the role of left M1 in language processing, we hypothesized that tDCS-induced upregulation of M1 will improve the performance of associating gestures and word comprehension.

## Materials and Methods

### Participants

Twenty-two right-handed healthy young adults (14 female; mean/SD/range age: 24/3/19–30 years; mean/SD/range Handedness score: 91/10/70–100) participated in the study. All were native German speakers and had no history of neurological or psychiatric disorders. The study was carried out in agreement with the Helsinki Declaration, and was approved by the ethics committee of the Charité Universitätsmedizin. Participants signed an informed consent form before participating.

### Study Design

**Figure 1:**
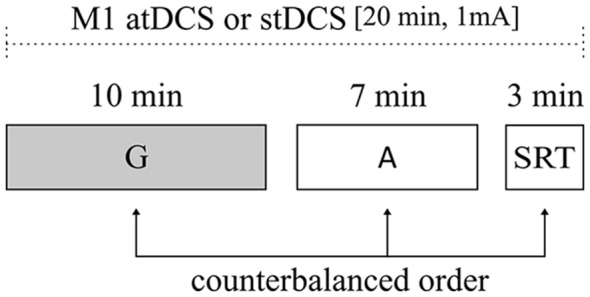
Illustration of experimental design. All subjects underwent atDCS and stDCS separated by at least 1 week, in a counterbalanced order. The duration of the main task (gestural task) and of control tasks [attentional load and simple reaction time (RT) tasks] are shown. Order of task presentation was counterbalanced between subjects. G, gestural task; A, attentional load task; SRT, simple RT task; atDCS, anodal transcranial direct current stimulation (tDCS); stDCS, sham tDCS.

In a within-subjects design, all young adults participated in two sessions where either anodal or sham tDCS was applied. Participants were blind to the stimulation condition. During the stimulation interval, participants were exposed to three different tasks that were presented in a counterbalanced order: gestural task, attention load task and simple RT task. Order of stimulation conditions was counterbalanced across subjects and sessions were separated by at least 1 week (Figure 1).

### Stimuli

The tasks were administered on the computer screen using the software Presentation (Neurobehavioral Systems^1^, version18.1).

#### Gestural Task

**Figure 2:**
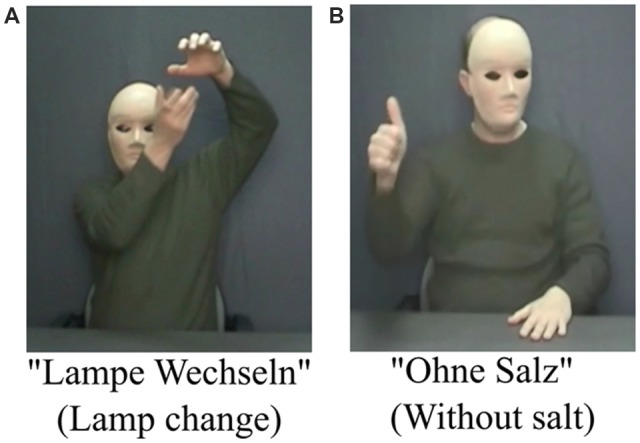
Examples of congruent and incongruent stimuli. **(A)** A gesture congruent with the word meaning and **(B)** a gesture incongruent with the word meaning.

The task was adapted from previous studies (Vainiger et al., 2014; Cohen-Maximov et al., 2015 for detailed description). Participants were exposed to a set of videos followed by set of written German words (prime, target). The duration of the task was 10 min. They were asked to choose whether the words describe the video or not, by clicking either the button “V” or “N” on the keyboard. They were instructed to use their right index finger and rest it on the “B” button between answers. Each trial began with the presentation of a fixation cross for 500 ms, followed by the 1,520 ms video clip (the prime). Gestures were grouped into instrumental and symbolic categories: instrumental gestures are those that imitate commonly known actions such as brushing teeth; symbolic gestures are those that carry figurative meaning such as “goodbye.” A third type of video consisted of landscape scenes such as an erupting volcano. All videos were followed by a short written German sentence comprising of a maximum of three words; the sentence was either congruent or incongruent in the preceding video. A total of 108 videos, grouped into two sessions with a 90 s break in-between, were presented. Of the 108 videos, 22 were instrumental congruent, 22 instrumental incongruent, 16 symbolic congruent, 16 symbolic incongruent, 16 landscapes congruent and 16 landscapes incongruent (Figure 2). RT was defined as main dependent variable. In addition, percentage of correct responses was assessed. Only correct answers with RT less than 2 s were included in the analyses.

#### Attentional Load Task

We adapted the Visual Attention—Flanker Compatibility Task Version 4 paradigm described elsewhere (Green and Bavelier, 2003). The duration of the task was 7 min. The task consisted of 12 practice trials and 96 experimental trials. Each trial in the attentional load task began with 500 ms of a central white fixation cross. Participants searched for two possible target shapes (“Square” or “Diamond”) among central non-target shapes. Participants were asked to indicate whether one of the Shapes was a “Square” or a “Diamond” by pressing “V” or “N” buttons on the keyboard. They were instructed to use their right index finger and rest it on the “B” button between answers. Attentional load was manipulated randomly between trials. Target position (1–6), target identity and distractor compatibility were counterbalanced when the trials were constructed. Two load conditions were presented, the low load (low competition condition), where the circle was composed of the target shape with no competing central shapes and the high load (high competition condition), where the circle was composed of the target shapes along with five competing shapes. A flanker appeared to the right or left of the circle in equal probabilities. The flankers were a “Square” or a “Diamond” and could be compatible with the target shape or not. Participants were instructed to ignore the flankers. The stimulus was presented for 100 ms. The interstimulus duration was 1,000 ms. RT and percentage of correct responses were assessed.

#### Simple Reaction Time Task

The duration of the task was 3 min. The task was adapted from Neurobehavioral Systems^1^; (e.g., Vieluf et al., 2017). Participants were instructed to click the “space” button on the keyboard as soon as they see a red square on the screen. The experiment consisted of eight practice trials and 100 experimental trials. The stimulus was presented for 500 ms with a rectangular distribution of inter-stimulus duration (between 1,000 ms and 2,000 ms). RT and percentage of correct responses were assessed.

### tDCS

Direct current stimulation was delivered through a battery-driven stimulator (neuroConn DC-Stimulator Plus, neuroCare Group GmbH, Munich, Germany) using two electrodes inserted in saline-soaked synthetic sponges. The anode (5 × 7 cm^2^) was centered over left sensorimotor cortex (left M1) and the reference electrode (10 × 10 cm^2^) was positioned over the contralateral (right) supraorbital region. Due to its larger size (10 × 10 cm^2^), the effect underneath the cathode is thought to be functionally less efficient (Nitsche et al., 2007; Nitsche and Paulus, 2011; Antal et al., 2017). Electrode positions were individually determined according to the 10-20 EEG system (active electrode centered over C3, reference over Fp2). The stimulation started with the beginning of the tasks. In the atDCS condition, stimulation was delivered continuously for 20 min (with 10-s fade in/out intervals) with a constant current of 1 mA. The duration of the stimulation was equal to the total duration of the three tasks. In the stDCS condition, stimulation was turned off after 30 s.

Before and after each stimulation condition mood ratings were assessed using the Positive and Negative Affect Schedule (PANAS; Watson et al., 1988). Participants rated their positive and negative affect (10 items each) on a scale ranging from 1–5, where higher values describe more positive or negative feelings, respectively. After completion of the second experimental session, participants were asked to retrospectively report the occurrence of adverse effects (pain, tingling, itching, burning, fatigue, tension, headache, discomfort) during stimulation in a standardized questionnaire (Poreisz et al., 2007). The questionnaire included the adverse effect with its corresponding intensity scale that ranges from 1–5 (with 1 as “very low” and 5 as “very high”).

### Statistical Analysis

IBM SPSS Statistics 24^2^ was used for statistical analyses. In order to test for differences between atDCS and stDCS, repeated-measures ANOVAs were performed, separately for all dependent variables. For the gestural task, congruency was added as within-subject factor. The interaction of congruency and stimulation effect was further assessed with subsequent ANOVAs conducted per congruency, in order to study the effect of stimulation on congruent and incongruent stimuli separately. In addition, an explorative analysis was conducted by adding the stimuli type (instrumental, symbolic, landscapes) as an additional factor and subsequent within-subjects *t*-tests were performed to compare RT of atDCS and stDCS in the three different incongruent stimuli types. Both percentage of correct responses and RT were analyzed separately as dependent variables for each task. Repeated-measures ANOVA were used for mood ratings with stimulation type (atDCS and stDCS) as within-subject factor. Generalized estimating equations were performed for adverse events to compare the frequencies of their occurrence under atDCS compared to stDCS. A two-sided significance level of α = 0.05 was used.

## Results

### Task Versions Validation

Two versions of the task were created. The similarity of the versions was validated in a pilot study. Ten native German speakers (6 females, Mean/SD age = 27.3/3.9) performed the two versions in a counterbalanced order. No difference in RTs was found between the two versions, version A (Mean/SD RT = 864/135 ms) and version B (Mean/SD RT = 809/113 ms), *U* = 41.5, *p* = 0.529. Likewise, no difference in percentage of correct responses was found between version A (Mean/SD percentage of correct responses = 91.7/3.2%) and version B (Mean/SD percentage of correct responses = 89.8/3.1%), *U* = 29.0, *p* = 0.123. In addition, gestures that could not be identified by participants because of their cultural specificity were omitted after task versions validation.

### Reaction Times (RTs)

In the gestural task, a 2 × 2 ANOVA showed that there was no significant main effect of stimulation condition on RT (*F*_(1, 21)_ = 2.41, *p* = 0.135). Interestingly, there was a significant interaction between stimulation condition and congruency, (*F*_(1, 21)_ = 5.21, *p* = 0.033, partial eta squared = 0.20). Subsequent ANOVAs conducted per congruency, showed that, for congruent stimuli, there was no significant difference in RT between atDCS and stDCS (*F*_(1, 21)_ = 0.013, *p* = 0.911). However, for incongruent stimuli, RT under atDCS was significantly faster compared to stDCS, (*F*_(1, 21)_ = 6.15, *p* = 0.022, partial eta squared = 0.23; Table 1). Moreover, there was no significant interaction between stimulation condition, congruency and stimuli type (*F*_(2, 20)_ = 2.87, *p* = 0.068). Subsequent exploratory within-subjects t-tests showed that for incongruent stimuli, RT of instrumental stimuli under atDCS (*M* = 871, SD = 99) was significantly faster compared to stDCS (*M* = 917, SD = 118); *t*_(21)_ = 2.9, *p* = 0.009 and RT of symbolic stimuli under atDCS (*M* = 879, SD = 115) was significantly faster compared to stDCS (*M* = 919, SD = 139); *t*_(21)_ = 2.2, *p* = 0.041. However, there was no significant difference in RT of landscape stimuli under atDCS (*M* = 825, SD = 103) compared to stDCS (*M* = 834, SD = 87); *t*_(21)_ = 0.552, *p* = 0.587.

**Table 1:** Summary of mean reaction times (RTs) and percentage of correct responses of all stimuli types in atDCS and stDCS for the gestural task, attentional load task and simple RT task.

	Reaction Time (ms)					Percentage of correct responses (%)				
	atDCS		stDCS			atDCS		stDCS		
	Mean	SD	Mean	SD	*p*	Mean	SD	Mean	SD	*p*
Gestural task										
Cong	876.0	101.8	877.4	92.1	0.911	0.90	0.04	0.89	0.05	0.739
Incong	859.5	97.1	891.8	108.9	**0.022**	0.97	0.03	0.96	0.04	0.288
Attentional load task	900.2	133.3	869.3	168.9	0.262	0.90	0.12	0.88	0.14	0.327
Simple reaction time task	267.5	20.0	267.8	23.1	0.926	0.99	0.01	0.98	0.02	0.056

*cong, congruent; incong, incongruent; SD, standard deviation; atDCS, anodal tDCS; stDCS, sham tDCS. Bold values refer to *p* < 0.05*.

**Figure 3:**
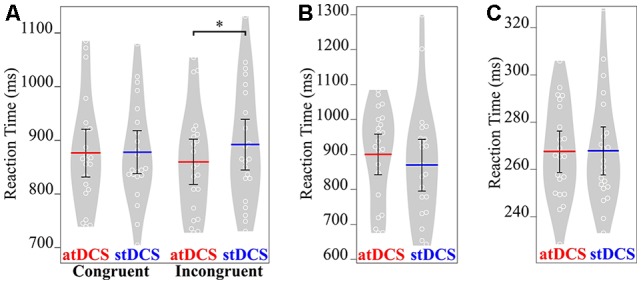
Mean RT (in ms) of the three tasks under both atDCS and stDCS. **(A)** Gestural task for congruent and incongruent stimuli. Only for incongruent stimuli, RT was significantly faster under atDCS compared to stDCS. **(B)** Attentional load and **(C)** simple RT task showed no significant difference in RT in atDCS compared to stDCS. Plots were constructed using BoxPlotR (Spitzer et al., 2014; http://shiny.chemgrid.org/boxplotr/). The white circles represent the individual data points, the red and blue lines represent the mean values across the group. Error bars represent 95% confidence interval. Violin plots show the distribution across subjects. *p < 0.05.

In both control tasks, there was no significant main effect of stimulation condition on RT (for attentional load task: (*F*_(1, 21)_ = 1.33, *p* = 0.262); for simple reaction time task: (*F*_(1, 21)_ = 0.01, *p* = 0.926; Figure 3).

### Percentage of Correct Responses

In the gestural task, a 2 × 2 ANOVA showed that there was no significant main effect of stimulation condition on percentage of correct responses (*F*_(1, 21)_ = 1.14, *p* = 0.298). There was no significant interaction between stimulation and congruency, (*F*_(1, 21)_ = 0.61, *p* = 0.807; Table 1). However, there was a significant main effect of congruency on percentage of correct responses (*F*_(1, 21)_ = 44.31, *p* < 0.001, partial eta squared = 0.68), indicating more correct responses for incongruent compared to congruent stimuli.

In both control tasks, there was no significant main effect of stimulation condition on percentage of correct responses (for attentional load task (*F*_(1, 21)_ = 1.01, *p* = 0.327), and for simple reaction time task (*F*_(1, 21)_ = 4.10, *p* = 0.056)).

### Mood Rating and Stimulation Side Effects

Table 2 shows mood ratings before and after stimulation for both atDCS and stDCS. Repeated-measures ANOVA showed that there was no significant difference in mood changes between atDCS and stDCS for both positive (*F*_(1, 21)_ = 1.623, *p* = 0.217) and negative (*F*_(1, 21)_ = 0.062, *p* = 0.806) affect.

**Table 2:** Participants’ mood ratings before and after stimulation.

	atDCS		stDCS	
	Mean	SD	Mean	SD
**Positive affect**	
Before	2.02	0.18	2.10	0.24
After	2.50	0.48	2.46	0.55
**Negative affect**				
Before	1.88	0.27	1.78	0.26
After	1.15	0.13	1.14	0.16

*atDCS, anodal tDCS; stDCS, sham tDCS*.

All subjects tolerated the stimulation with only few subjects reporting adverse effects; Table 2 shows the number of participants reporting each adverse effect in the corresponding condition. From participants who reported adverse effects in atDCS, more than 50% scaled the intensity as less than 2 (out of 5). Only 4 of 22 subjects noticed a difference and reported a slightly stronger tingling sensation due to atDCS, indicating an overall efficient placebo condition. Generalized estimating equation did not show any significant differences in atDCS compared to stDCS (*p* values > 0.106). PANAS and adverse events (AEs) of tDCS are presented in Tables 2, 3 respectively.

**Table 3:** Number of participants who reported adverse effects (total *N* = 22).

	atDCS	stDCS
Pain	5	4
Tingling	2	5
Itchiness	7	2
Burning	4	2
Fatigue	4	2
Tension	–	4
Loss of concentration	3	2
Discomfort	1	–

## Discussion

This study assessed the effect of excitatory atDCS over left M1 on gestural-verbal integration. We found that atDCS led to significantly faster correct answers compared to stDCS, but only for incongruent and not for congruent associations. Performance on attentional load and simple reaction time tasks was not affected by the stimulation.

### Neural Correlates of Gestural-Verbal Integration

In the present study, we observed faster responses during atDCS compared to stDCS in the gestural-verbal task. This result is consistent with previous studies that showed a tDCS-induced improvement in cognitive functions (Radman et al., 2018; Wang et al., 2018; Yang et al., 2018). Further studies investigated gestural-verbal integration and gesture processing using atDCS over frontal (Cohen-Maximov et al., 2015; Schulke and Straube, 2017) and parietal cortices (Bianchi et al., 2015). Results showed that stimulation of frontal area decreased RTs of processing gestural-verbal associations (Cohen-Maximov et al., 2015; Schulke and Straube, 2017). Similarly, stimulation of the left parietal cortex improved motor test scores in apraxia patients (Bianchi et al., 2015). Taking together, atDCS over frontal and parietal cortices has been shown to improve both gestural-verbal integration and gesture comprehension. More recently, Schulke and Straube (2017) applied tDCS during a speech-gesture semantic task. They found that inhibitory cathodal tDCS (ctDCS) decreased task performance and atDCS enhanced it. As for motor cortex, previous studies investigated the role of motor cortex in language processing using atDCS (Meinzer et al., 2016; Branscheidt et al., 2018). For example, Meinzer et al. (2016) applied atDCS over left motor cortex twice daily at the beginning of a naming therapy and suggested that stimulating this area enhanced naming ability of patients with post-stroke aphasia. Branscheidt and colleagues followed up on Meinzer et al.’s (2016) findings by also applying atDCS over left motor cortex showing that atDCS improved lexical decision accuracy selectively for action-related words and not for object-related ones (Branscheidt et al., 2018). More specifically, we observed faster responses in atDCS compared to stDCS for incongruent associations. This indicates the involvement of motor areas in the processing of language and gesture when the information conveyed by the gesture does not describe the information conveyed by language. Our results corroborate previous studies showing activation of left IFG and its adjacent motor areas during the processing of incongruent speech-gesture associations (Willems et al., 2007; Green et al., 2009; Kircher et al., 2009). For instance, Willems et al. (2007) measured brain activity using fMRI during performance of a gestural-verbal semantic task in healthy young subjects. They showed that premotor cortex was activated only when the gesture did not match the word. More recently, studies have shown that IFG is involved in gestural-verbal integration in case of semantic conflict (Willems et al., 2007; Green et al., 2009; Zhao et al., 2018). In addition, IFG and premotor cortex are anatomically connected and are both activated in gestural-verbal integration (Marstaller and Burianova, 2015). It is thus possible that activation of one region is affected by the activation of the other, creating an extended network sensitive to the congruency of gestural-verbal association. However, negative findings were also reported: for example, Siciliano et al. (2016) applied anodal tDCS over left IFG during language learning and found no difference between anodal and sham conditions when words were coupled with gestures. In contrast to our study, the authors only included gestures that were congruent with the presented word in their task.

Our subsequent explorative results showed that faster responses under atDCS compared to stDCS were found only for gestural stimuli (instrumental and symbolic), and not for landscape stimuli. In accordance with previous studies suggesting that motor and premotor cortices are activated only in action-related gestures like instrumental gestures (He et al., 2018a), and that left frontal cortex is specifically implicated in the understanding of symbolic gestures (Rapp et al., 2007, 2012; Straube et al., 2013), our initial hypothesis was that only instrumental gestures would be affected. However, our results are in line with previous studies suggesting no difference in the processing of instrumental and symbolic gestures (He et al., 2015, 2018b), indicating that both gesture types may be supported by left motor activity. Nevertheless, these findings should be interpreted with caution due to the relatively small number of stimuli within the stimulus categories.

### Involvement of M1 in Cognitive Control Mechanism

In line with our results, tDCS has been previously implicated in modulating task performance in incongruent associations; this hypothesis was investigated using paradigms that include an interference effect like the flanker task and Stroop task (Ouellet et al., 2015; Zmigrod et al., 2016). For instance, a recent study applied ctDCS during a flanker task to examine the effect of stimulation on performance and showed that ctDCS led to slower RTs when the flanker was incongruent with the target (Zmigrod et al., 2016). Thus, tDCS was shown to affect performance of incongruent stimuli associations. Further, Botvinick et al. (2001) suggested that the brain responds to interference (in this case incongruent associations) by implementing a cognitive control mechanism. It was suggested that the cognitive control mechanism is not defined as a simple task, related to one brain region, but rather it is recognized as a cascade of distinct control types performed by distinct brain regions. For instance, in a brain imaging study, dorsolateral prefrontal cortex (DLPFC) was shown to engage its control through the top-down modulation of task-dependent information processing in M1, also referred to as sensory control (Koechlin et al., 2003). Based on these studies, the improvement of subjects’ ability to detect incongruent gestural-verbal associations, or in other words cases of semantic conflicts, might result from increased cognitive control mechanisms following modulation of brain circuitry underlying sensory control in M1 region. In addition, a previous study used high-definition tDCS over DLPC or over M1 during a flanker task and showed that tDCS over M1 region did not show any significant effect on conflict adaptation effect which is a hallmark of cognitive control mechanism (Gbadeyan et al., 2016). This result complements our results in assuming that the effect we found might be specific to the gestural-verbal task, since the flanker task used in the previous study does not include a motor component. Moreover, given that atDCS did not affect attentional load task, which is a classical conflict task, this result further supported our assumption that the cognitive control mechanism generated by the M1 might be specific to motor action, i.e., gestural-verbal integration and rather than simply affecting attention. It might be interesting to compare our results with DLPFC stimulation of the same task, to test direct cognitive control effects on gestural-verbal associations. Taken together, our findings suggest that atDCS over left M1 might have affected cognitive control mechanisms, leading to better performance in the detection of incongruent associations. Regardless of stimulation condition, we found a significantly higher accuracy for incongruent associations compared to congruent ones. Previous studies observed a similar pattern (Proverbio et al., 2014, 2015; Kelly et al., 2015), possibly due to the fact that semantic matching decision may be easier when it is obviously violated by incongruent associations.

In addition, our results go along with a recent study that used tDCS over right temporo-parietal junction followed by transcranial magnetic stimulation application over left M1. Participants were instructed to perform an action while observing either a congruent or incongruent action, while motor evoked potentials (MEPs) were elicited. Results showed that only during observation of incongruent actions, MEPs were significantly higher under atDCS compared to stDCS. This study suggested that up-regulation of the appropriate motor action leads to suppression of the congruency effect, possibly via interaction between the temporo-parietal junction and M1 (Bardi et al., 2017). The process of action observation and action execution in M1 region is related to gestural-verbal integration in humans, a process that was found to be related to mirror neuron system (for review Rizzolatti et al., 2014). Despite the fact that our paradigm included gesture observation and not production, our findings suggest that atDCS may have improved gestural-verbal integration by facilitating mirror neuron system mapping activity.

Several limitations should be considered when interpreting the results of the present study. First, the sample size was relatively modest and larger future studies be conducted to confirm these results. Second, we used tDCS, a stimulation device with low spatial resolution (Polania et al., 2018). To infer specificity of left M1 for gestural-verbal integration, a control tDCS site would have to be included. Nevertheless, specificity of left M1 atDCS for the gestural task was demonstrated by the absence of effects on the control tasks. Finally, the sequential presentation of gesture and words might be considered less natural than the integration of auditory input and visual gestural information, so future studies should include paradigms with auditory presentation of words, to more closely resemble the natural setting of language and gesture processing.

## Conclusions and Outlook

In sum, we found that enhancing left M1 improves semantic processing by specifically enhancing performance of incongruent stimuli, possibly mediated by facilitation the action perception sensitivity of the MNS. In addition, the excitatory effect of tDCS on M1 might have increased its cognitive control potential, leading to lower interference. Future studies should in more detail investigate the neural correlates of semantic congruency with tDCS and EEG exploring also the interaction between the M1 and other brain regions involved in cognitive control like the DLPFC (Koechlin et al., 2003; Gbadeyan et al., 2016), or brain regions know to closely interact with the MNS like the IFG (for review Buccino et al., 2004; Jeon and Lee, 2018). In the clinical context, tDCS effects on speech and gesture processing may be relevant for patients with schizophrenia who suffer from severe deficits in speech and gesture processing (Schulke and Straube, 2018).

## Author Contributions

DH, AF and DA designed the research. DH and DA performed the research. DH and DA analyzed the data. DH, AF and DA wrote the article.

## Conflict of Interest Statement

The authors declare that the research was conducted in the absence of any commercial or financial relationships that could be construed as a potential conflict of interest.

## Footnotes

^1^https://www.neurobs.com/

^2^www-03.ibm.com/software/
